# Optimization of Thermoelectric Nanoantenna for Massive High-Output-Voltage Arrays

**DOI:** 10.3390/nano14131159

**Published:** 2024-07-07

**Authors:** Mohamad Khoirul Anam, Yudhistira Yudhistira, Sangjo Choi

**Affiliations:** 1 Research Center for Testing Technology and Standard, National Research and Innovation Agency (BRIN), South Tangerang 15314, Indonesia; moha048@brin.go.id; 2 School of Electronics Engineering, Kyungpook National University, Daegu 41566, Republic of Korea; yudhis89@knu.ac.kr

**Keywords:** thermoelectric nanoantenna, nano-thermocouple, Seebeck coefficient

## Abstract

Thermoelectric nanoantennas have been extensively investigated due to their ability to directly convert infrared (IR) radiation into direct current without an additional rectification device. In this study, we introduce a thermoelectric nanoantenna geometry for maximum output voltage (*V*_oc_) and propose an optimal series array configuration with a finite number of antennas to enhance the *V*_oc_. A finite and open-ended SiO_2_ substrate, with a thickness of a quarter-effective wavelength at a frequency of 28.3 THz, is used to generate standing waves within the substrate. An array of antennas is then positioned optimally on the substrate to maximize the temperature difference (∆T) between hot and cold areas, thereby increasing the average *V*_oc_ per antenna element. In numerical simulations, a linearly polarized incident wave with a power density of 1.42 W/cm^2^ is applied to the structure. The results show that a single antenna with the optimum geometry on a substrate measuring 35 µm × 35 µm generates a ∆*T* of 64.89 mK, corresponding to a *V*_oc_ of 1.75 µV. Finally, a series array of 5 × 6 thermoelectric nanoantennas on a 150 µm × 75 µm substrate including measurement pads achieves an average ∆*T* of 49.60 mK with a total *V*_oc_ of 40.18 µV, resulting in an average *V*_oc_ of 1.34 µV per antenna element and a voltage responsivity (*β_v_*) of 0.77 V/W. This value, achieved solely by optimizing the antenna geometry and open-ended substrate, matches or exceeds the *V*_oc_ and *β_v_* of approximately 1 µV and 0.66 V/W, respectively, from suspended thermoelectric antenna arrays over air cavities. Therefore, the proposed thermoelectric nanoantenna array device, characterized by high stability and ease of fabrication, is suitable for manufacturing massive nanoantenna arrays for high-output IR-DC energy harvesters.

## 1. Introduction

Infrared (IR) radiation emitted by artificial sources such as heating devices covers a wide range of IR wavelengths. However, a large portion of this IR energy is typically unused as thermal waste energy [1,2,3]. This has motivated the development of systems for capturing IR waste energy and converting it into another form of energy, such as electricity. Among various methods, thermoelectric nanoantennas represent highly cost-effective devices for directly converting IR radiation into electricity without requiring additional rectification devices such as metal–insulator–metal and metal–oxide–semiconductor-based nanoantennas [4,5].

Generally, the antenna structure in thermoelectric devices consists of a dipole nanoantenna as an efficient receiver for capturing external IR radiation. The center of this nanoantenna is connected with a nano-thermocouple that converts the captured IR radiation into an open-circuit voltage (*V*_oc_). The electrical length of the nanoantenna typically matches the half-effective wavelength of the incident IR wave, resulting in a strong current at the center [6,7,8]. This current increases the antenna temperature at its center due to the kinetic energy from interactions between charge carriers and quasi-particles from the antenna element [9,10,11,12]. The resulting temperature gradient causes the thermally excited electrons from the antenna to travel with higher energy along the nano-thermocouple, resulting in the generation of *V*_oc_ [13]. The *V*_oc_ can be calculated by multiplying the relative Seebeck coefficient (Δ*S*) of the nano-thermocouple and the temperature difference between hot and cold areas (Δ*T*) [14,15,16]. Although materials such as telluride-based materials and carbon nanotubes with high Seebeck coefficients can be used for the thermocouple in the thermoelectric nanoantenna design, we aimed to find the optimum nanoantenna using a metal-based thermocouple [17,18,19].

Thermoelectric nanoantennas with metal-based thermocouples mounted on a grounded substrate were used for IR-DC conversion [20,21]. However, this structure provided a low *V*_oc_ as only one antenna element contributed to the total *V*_oc_. To achieve a higher *V*_oc_, several thermoelectric nanoantennas were electrically connected in series [20,22,23,24]. Specifically, an array concept was realized using a series connection of dipole-shaped nanoantennas integrated with a single metal thermocouple composed of Ni, achieving a *V*_oc_ of 3.25 µV with a ∆*T* of 15.5 mK between the hot and cold junctions [22]. Although this structure incorporated a large number (>400) of nanoantennas coupled with thermocouples, the average *V*_oc_ per antenna element (7.38 nV) was too low due to a small Seebeck coefficient difference associated with the single metal thermocouple.

To increase the average *V*_oc_, researchers have proposed a nanoantenna array structure combined with a bimetal thermocouple to achieve a higher Seebeck coefficient difference. For example, Szakmany et al. designed a dipole nanoantenna array combined with a bimetal thermocouple (Au-Pd), achieving an improved average *V*_oc_ of 0.12 µV due to the higher difference in the Seebeck coefficient [20]. The *V*_oc_ was further improved by designing an antenna array structure on a thermally insulated area to prevent heat transfer from the antenna center [23,25,26]. To implement this concept, Szakmany et al. suspended a large array of thermoelectric nanoantennas (>200 elements) in a series connection over an air-filled cavity, resulting in a *V*_oc_ of 200 µV, with an average *V*_oc_ of 1 µV per antenna [23]. However, the use of a single metal thermocouple with a low Seebeck coefficient difference limited the *V*_oc_, despite a high temperature difference in the array. Bimetal versions of similar air-cavity-based arrays with 48 antennas have been reported, but only the relative differences in *V*_oc_ depending on the atmospheric pressure, without absolute *V*_oc_ levels from the arrays, were presented [27]. To the best of the authors’ knowledge, a single antenna utilizing a bimetal thermocouple and an air-cavity demonstrated a high *V*_oc_ of 38 µV, but such a high *V*_oc_ per antenna from its array has not yet been reported [21]. Recently, Anam et al. designed a bimetal thermoelectric nanoantenna on a grounded and open-ended SiO_2_ substrate with a quarter-wavelength thickness and increased the *V*_oc_ to 2 µV [28]. The SiO_2_ substrate acted as a cavity and maximized the field at the antenna center through standing waves launched inside the substrate [28]. This device geometry was also more durable than air-cavity-based nanoantennas. Therefore, it is worthwhile to investigate the optimum thermoelectric nanoantenna geometry on a SiO_2_-based cavity and its array design to maximize the *V*_oc_ from thermoelectric nanoantenna devices.

In this study, we investigated the optimal geometry for a bimetal thermoelectric nanoantenna to achieve the maximum *V*_oc_ by optimizing the coupling between the antenna’s electric field and the standing wave in a grounded and open-ended SiO_2_ cavity. Based on the optimum single antenna geometry, we designed nanoantenna array structures, optimizing the vertical and horizontal distances between the antennas to maximize the *V*_oc_. Using these optimum distances, a 5 × 6 thermoelectric nanoantenna array structure achieved a *V*_oc_ of 40.18 µV, with an average *V*_oc_ of 1.31 µV per antenna and a voltage responsivity (*β_v_*) of 0.77 V/W. The increased average *V*_oc_ was attributed to the enhanced field at the antenna elements due to the standing wave in the SiO_2_ cavity. The average *V*_oc_ and *β_v_* were higher than the *V*_oc_ of 1 µV per antenna and *β_v_* of 0.66 V/W from the existing thermoelectric nanoantenna array suspended over an air cavity [23]. The proposed thermoelectric nanoantenna array structure mounted on the SiO_2_ cavity provided a superior *V*_oc_ and greater fabrication stability, rendering it promising for the development of high-output IR-energy-harvesting devices or sensors.

## 2. Simulation Methods

A numerical study was conducted to calculate the current density and temperature difference (∆*T*) between the hot and cold junctions of a thermoelectric nanoantenna using heat transfer modules in COMSOL Multiphysics 5.0. Ti and Ni were used as metallic traces for the antenna and thermocouple, respectively. SiO_2_ and Al were used for the substrate and reflector, respectively. The relative permittivity (ε) and conductivity (σ) of the materials in the far IR region were calculated as $\varepsilon =n^{2}-k^{2}$ and $\sigma =\varepsilon_{0}\times \varepsilon_{2}\times \omega$, respectively. Here, n and k denote the real and imaginary parts of the refractive index, respectively; and $\varepsilon_{0},\;\varepsilon_{2}$, and ω are the vacuum permittivity, the imaginary part of the relative permittivity, and the angular frequency, respectively [29,30,31]. For heat transfer calculations, the thermal conductivity, heat capacity, and mass density were extracted from a previous study [32]. Overall, the scope of the simulation was based on microscopic or continuum-level properties of the materials instead of atomistic modeling including the topological heterostructure in the nanoantenna structure [33]. Although the junctions between the thermocouple and the nanoantenna could be analyzed more accurately by atomistic modeling, this was beyond the scope of this study.

**Figure 1:**
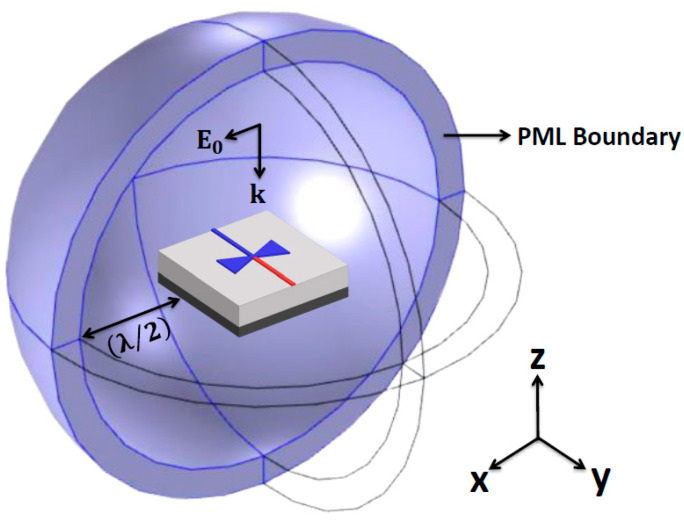
Schematic of the thermoelectric nanoantenna simulation, where an incident electric field (*E*_0_) aligned with the antenna axis and propagated in the negative *z*-direction. The simulation boundary was defined as a perfectly matched layer (PML).

In the simulation, the device was illuminated by a linearly polarized plane wave propagating along the negative *z*-direction, with the simulation boundary set by a perfectly matched layer (PML) with a thickness of (λ/2) to absorb outgoing waves without reflection, as shown in Figure 1. The vector component of the incident electric field (*E*_0_) with a power density of 1.42 W/cm^2^ was aligned with the antenna axis for the highest current excitation at the antenna center. To calculate the temperature difference (Δ*T*), heat transfer analysis was used by employing a convective boundary condition with a heat transfer coefficient of 5 W/m^2^·K from metal to free space [34]. The Δ*T* of the structure was calculated using Δ*T* = *T*_hot_ − *T*_cold_, where *T*_hot_ and *T*_cold_ are the temperatures at the hot and cold junctions, located at the antenna center and ends of the nano-thermocouple, respectively. Finally, using the Seebeck coefficients of Ti (*S*_Ti_) and Ni (*S*_Ni_) of 7.19 and −19.5 µV/K [35], respectively, Δ*T* was translated into *V*_oc_ using Δ*S* × Δ*T*, where Δ*S* is *S*_Ti_ − *S*_Ni_, the Seebeck coefficient difference between Ti and Ni [36]. It is noted that the Δ*S* of the bimetal junction in the thermocouple can vary compared to the values from their bulk materials due to the nanometer dimensions [37,38,39]. In simulations, the bulk values were used based on the reported measured data where a Pd-Ni junction of 75 nm^2^ using Ni showed a similar Δ*S* compared to the bulk value, and a Ti-Ni-junction-coupled nanoantenna demonstrated good agreement between the measured *V*_oc_ and the simulated values using bulk Seebeck coefficients [28,38]. Nevertheless, the direct experimental verification of Δ*S* for the nanometer Ti-Ni junction will be worth investigating.

## 3. Results and Discussion

First, we investigated the optimal geometry for a single thermoelectric nanoantenna to achieve the maximum *V*_oc_ in a given substrate. Using this optimal geometry, we arranged the antennas into a finite-series array structure to enhance the *V*_oc_. To this end, we tuned the array parameter, such as the nano-thermocouple length (*L*_t_), antenna pitch size (*P*), and distance from the antenna to the substrate boundary (*G*) of the antennas in a finite SiO_2_ substrate. Finally, a 5 × 6 finite array with 30 antennas was designed using the optimum array parameters, and the device’s *V*_oc_ and *β_v_* were calculated and analyzed.

### 3.1. Optimization of Single Thermoelectric Nanoantenna Geometry

**Figure 2:**
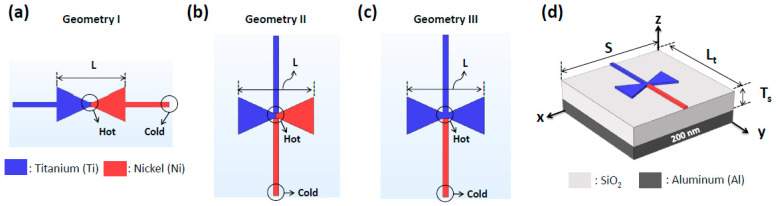
Schematics of the single antennas with different geometries. (**a**) Bimetal antenna connected in parallel with a nano-thermocouple (geometry I), (**b**) bimetal antenna connected perpendicularly with a nano-thermocouple (geometry II), and (**c**) single metal antenna connected perpendicularly with a nano-thermocouple (geometry III). (**d**) Schematic of a single thermoelectric nanoantenna (geometry III) mounted on a SiO_2_-grounded substrate.

First, a single antenna was designed to achieve a high Δ*T* at a resonant frequency of 28.3 THz (wavelength of 10.6 µm). We used a bowtie-shaped nanoantenna to capture IR radiation instead of a dipole-shaped nanoantenna due to its larger effective area, resulting in a higher current at the antenna center. A pair of bimetal Ni-Ti was chosen as a nano-thermocouple to convert the Δ*T* into *V*_oc_. Three thermoelectric nanoantenna geometries were devised, as shown in Figure 2a–c, with the hot and cold junctions located at the antenna center and ends of the nano-thermocouple, respectively. In geometry I, the nano-thermocouple was connected to the middle of the two ends of the bimetal (Ni-Ti) bowtie nanoantenna in parallel. In geometry II, the same bimetal nanoantenna and thermocouple were used, but they were connected perpendicularly at the center of the bowtie structure. Geometry III was similar to geometry II with the antenna changed to a single metal (Ti) bowtie antenna [28]. In the implementation, the antenna was directly mounted on top of an open-ended SiO_2_ substrate backed with an Al reflector, as shown in Figure 2d. In all the geometries, the antenna length (*L*) was set as 1225 nm for resonance at 28.3 THz, and the substrate size (*S*) was set as 35 μm, supporting the TM_40_ mode in the substrate for constructive coupling at the antenna center [28]. The length (*L*_t_) and width of the thermocouple were set as 35 μm and 70 nm, respectively, and the thickness of all the metallic structures, including the antenna and thermocouple, was 60 nm. The thickness (*T*_s_) of the SiO_2_ substrate was set as 1.2 µm, a quarter of the effective wavelength inside SiO_2_ to achieve constructive coupling between the incident and reflected waves at the antenna. Lastly, a 200 nm thick Al reflector, considerably thicker than the skin depth of Al at 28.3 THz and fabricable using a conventional e-beam evaporation method, was used to perfectly reflect the incident wave without any leakage [28].

**Figure 3:**
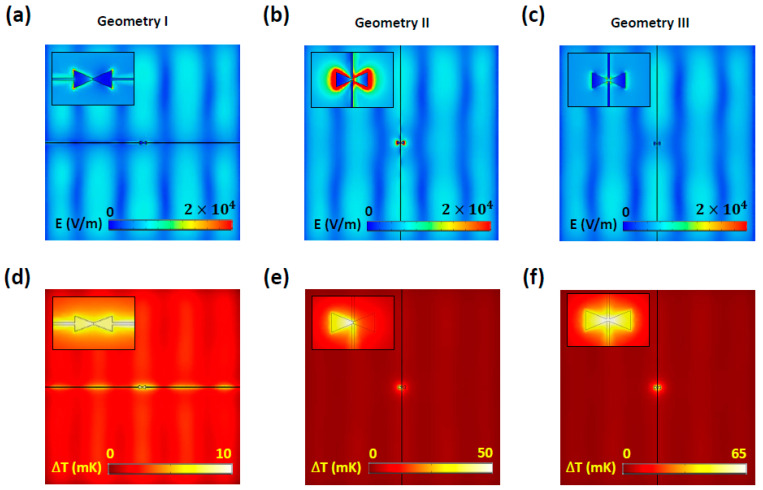
Electric field and temperature distribution on the *x*–*y* plane in the middle of the antenna at 28.3 THz for (**a**,**d**) geometry I, (**b**,**e**) geometry II, and (**c**,**f**) geometry III.

To determine the optimal antenna geometry for the highest *V*_oc_, we calculated the electric field and temperature distribution on the *x–y* plane in the middle of the antennas. Figure 3a–c show the electric field distribution with magnified views near the antennas (insets). In all cases, standing waves with maximum magnitude at the center of the substrate were generated due to the finite-sized and open-ended SiO_2_. In Figure 3a, the inset shows that the electric field of geometry I was distributed between the antenna and thermocouple because the ends of the antenna were connected to the thermocouple in parallel. Strong fields at the sharp tips of the antenna indicated that resonance from the antenna length was only maintained at small spots. This parallel connection between the antenna and thermocouple could not effectively confine and boost the fields near the thermocouple junction located at the antenna center. In contrast, as shown in Figure 3b,c, the antennas perpendicularly connected to the thermocouple at the antenna center exhibited considerably stronger fields near the antenna center. In both cases, the resonance from the finite-sized antenna was less perturbed compared with that of geometry I. In Figure 3b, representing geometry II, the highest field was observed near the antenna, likely due to the higher conductivity of Ni compared with Ti used in geometry III.

Figure 3d–f show the temperature distribution on the *x*–*y* plane for the three geometries. In geometry I, the distributed field along the antenna and thermocouple caused heat to spread from the antenna to the thermocouple, reducing the Δ*T* between the hot and cold junctions, as shown in Figure 3d. Figure 3e shows that geometry II maintained a ∆*T* reaching up to 50 mK, due to the higher electric fields near the antenna. However, the bowtie arm with Ni exhibited a much lower temperature, attributable to its higher thermal conductivity compared with that of Ti. Lastly, geometry III exhibited the highest ∆*T* approaching 65 mK, despite having lower fields near the antenna compared with geometry II. This highest temperature in geometry III can be attributed to the lower thermal connectivity of Ti. In conclusion, to maximize the *V*_oc_ of a thermoelectric antenna integrated with a bimetal (Ni-Ti) thermocouple, the construction of a single metal (Ti) nanoantenna with lower electrical and thermal conductivity between two metals (Ni and Ti) and its perpendicular connection to the bimetal thermocouple are necessary.

**Figure 4:**
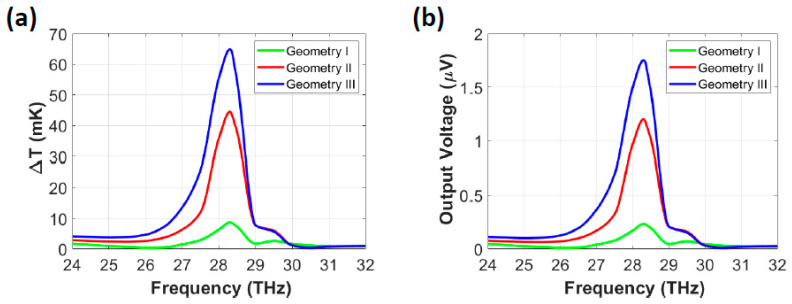
(**a**) Δ*T* and (**b**) *V*_oc_ for three single thermoelectric nanoantenna geometries as a function of frequency.

Figure 4a shows the Δ*T* of the three geometries of the single thermoelectric nanoantenna as a function of frequency. Geometry III provided the highest Δ*T* of 64.89 mK at 28.3 THz, followed by geometry II and geometry I, with values of 44.51 mK and 8.65 mK, respectively. Figure 4b illustrates that the *V*_oc_ values of the three geometries were directly proportional to Δ*T*, with geometry III providing the maximum *V*_oc_ of 1.75 µV at 28.3 THz. Based on the simulation results, geometry III was chosen for array designs to achieve the highest *V*_oc_, as discussed in the following sections.

### 3.2. Finite Array Structure

#### 3.2.1. Optimization of Nano-Thermocouple Length *L*_t_

**Figure 5:**
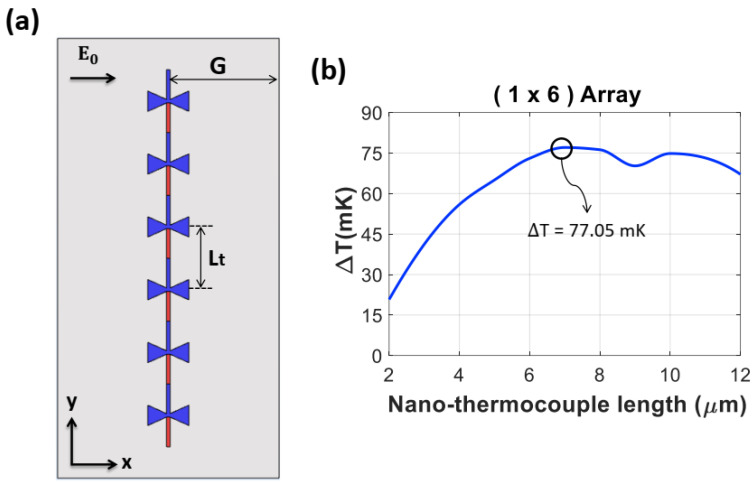
(**a**) Schematic of the 1 × 6 array of thermoelectric nanoantennas on the *x*–*y* plane. The nano-thermocouple length (*L*_t_) is the distance between two antennas along the vertical axis (*y*-axis). (**b**) Average ∆*T* at a resonant frequency of 28.3 THz as a function of *L*_t_.

We first designed a finite array of 1 × 6 antennas connected in series along the *y*-axis (Figure 5a) and tuned the nano-thermocouple length (*L*_t_), the distance between the centers of adjacent antennas along the *y*-axis, to achieve the maximum ∆*T* at a resonant frequency of 28.3 THz. We maintained a distance from the antenna center to the substrate boundary (*G*) as 17.5 µm, half of the *S* (35 µm) used in the single antenna geometry. Figure 5b shows the average ∆*T* at a resonant frequency of 28.3 THz as a function of *L*_t_, indicating a maximum ∆*T* of 77.05 mK at an *L*_t_ of 7 µm.

**Figure 6:**
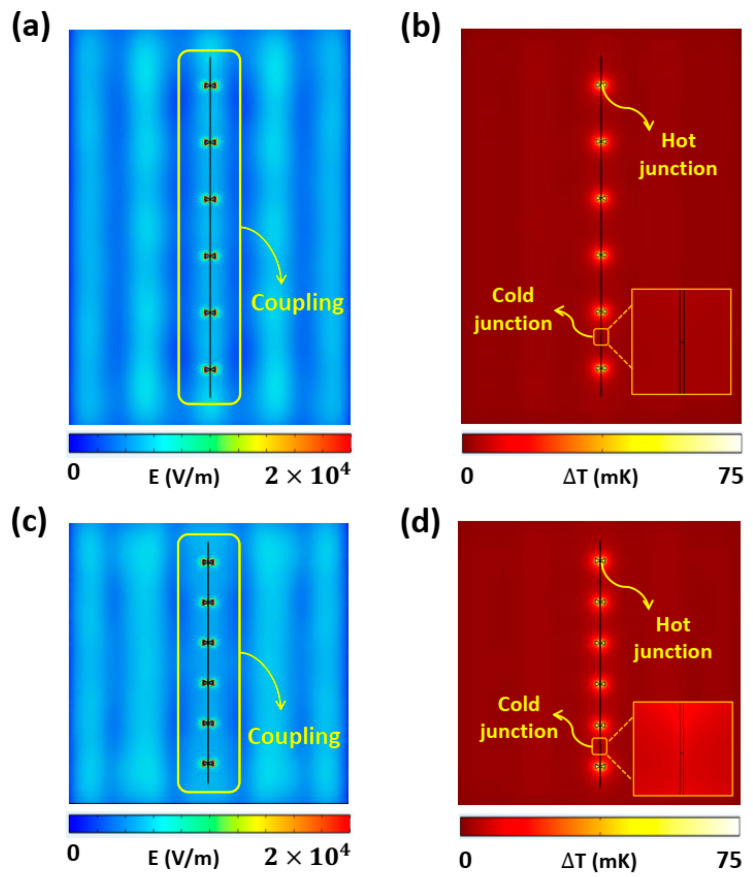
Electric field and temperature distributions on the *x*–*y* plane in the middle of the antennas in the 1 × 6 antenna array at 28.3 THz. Figures (**a**,**b**) and (**c**,**d**) correspond to *L*_t_ = 7 µm and *L*_t_ = 5 µm, respectively.

To gain a physical understanding of the dependence of *L*_t_ on ∆*T*, Figure 6 shows the electric field and temperature distributions on the *x*–*y* plane in the middle of the antennas in the 1 × 6 antenna array at 28.3 THz for two cases: *L*_t_ = 7 µm and 5 µm. In both cases, the antennas in the array were constructively coupled to the standing wave at the center of the substrate, as shown in Figure 6a,c. Figure 6b,d confirm that the temperatures were elevated at all the antenna elements (hot junctions) compared with the ends of the nano-thermocouples (cold junctions). Although coupling occurred uniformly in both the hot and cold junctions, the optimal distance (*L*_t_ = 7 µm) between the antennas effectively suppressed the field at the ends of the thermocouple, resulting in uniformly lower temperatures at the cold junctions compared with those at the hot junctions, as shown in Figure 6b (inset). Consequently, the highest average ∆*T* was generated across all antennas when the *L*_t_ was 7 µm. Conversely, when the *L*_t_ was 5 µm, Figure 6d (inset) demonstrates that the temperature at the cold junctions was not sufficiently suppressed, resulting in a lower ∆*T*. This investigation revealed that the length of the thermocouple (*L*_t_) connected to the nanoantenna must be optimized for suppressing the field strength at the cold junctions, even though all the antennas were constructively coupled to the standing wave generated by the SiO_2_ cavity.

#### 3.2.2. Optimization of Antenna Pitch (*P*) and Boundary Distance (*G*)

**Figure 7:**
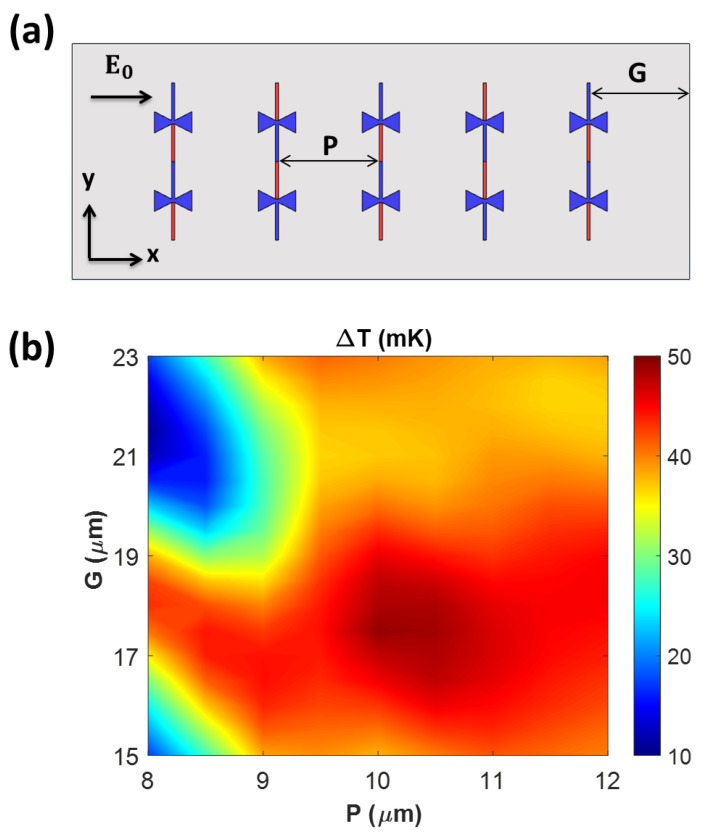
(**a**) Schematic of a 5 × 2 array of thermoelectric nanoantennas, indicating the antenna pitch (*P*), the distance between the antenna center in the horizontal axis (*x*-axis), and the distance between the antenna near the boundary and substrate boundary (*G*). (**b**) Contour plot of Δ*T* with different *P* and *G* values.

In addition to optimizing the *L*_t_ along the *y*-axis, we optimized the array geometry along the *x*-axis, including the antenna pitch (*P*) and the distance between the antenna near the boundary and substrate boundary (*G*) to achieve the maximum average ∆*T* at 28.3 THz. In the optimization process, the *P* and *G* were simultaneously varied in a finite 5 × 2 array with a condition (*G* ≥ 15 µm), providing a sufficiently large area for five 1 × 2 arrays to be mounted stably, as shown in Figure 7a. All antennas were directed to the *x*-axis, parallel to the polarization of the incident wave, and the antennas in 1 × 2 arrays were arranged with *L*_t_ = 7 µm along the *y*-axis. Five pairs of 1 × 2 arrays were then arranged along the *x*-axis with different *P* and *G* values varying by 0.5 µm. Figure 7b presents the average ∆*T* distribution of the finite 5 × 2 array as a function of *P* and *G* at 28.3 THz. Specifically, *P* = 10 µm and *G* = 17.5 µm provided the maximum ∆*T* of 49.30 mK, while the minimum ∆*T* of 10.95 mK was observed at *P* = 8 µm and *G* = 21.5 µm. Using the relationship (*S* = 4*P* + 2*G*) between the substrate size along the *x*-axis (*S*), *P*, and *G*, both cases maintained the same *S* of 75 µm, which supported the TM_90_ mode in the substrate according to [28].

**Figure 8:**
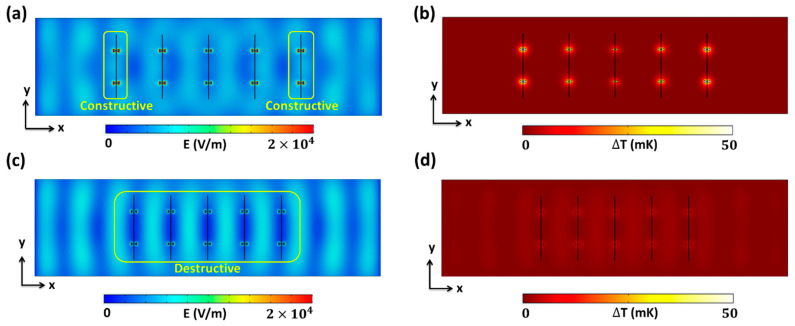
Electric field and temperature distribution of the 5 × 2 antenna array on the *x*–*y* plane in the middle of the antennas at 28.3 THz. (**a**,**b**) *P* = 10 µm, *G* = 17.5 µm; and (**c**,**d**) *P* = 8 µm, *G* = 21.5 µm.

To investigate the coupling behavior between the antenna’s electric fields and standing waves from the substrate in two extreme cases, we analyzed the electric field and temperature distributions on the *x*–*y* plane, as shown in Figure 8. Figure 8a,c reveal that the constructively and destructively coupled positions of the standing waves in the substrates were maintained in both cases, with only magnitude variations. The variation in field strength in the antennas was due to changes in the array positions along the *x*-axis. Specifically, Figure 8a, which corresponds to the maximum ∆*T* with *P* = 10 µm and *G* = 17.5 µm, shows that five 1 × 2 arrays were positioned along the *x*-axis to maximize the averaged field strength in the antennas. Due to the destructively coupled standing wave at the substrate center from the TM_90_ mode, the optimum *P* and *G* values located two arrays at constructively coupled positions near the boundaries, while one array at the center was located in a destructively coupled position. Conversely, in the minimum ∆*T* case with *P* = 8 µm and *G* = 21.5 µm, all the arrays were positioned at destructively coupled positions of the standing wave in the substrate. This coupling variation along the *x*-axis was translated into distinct ∆*T* differences between the two cases, as shown in Figure 8b,d. The higher coupling from the *P* = 10 µm and *G* = 17.5 µm case resulted in a highly elevated ∆*T* in all the antenna elements, as shown in Figure 8b. In addition, the non-uniform ∆*T* distribution in the five 1 × 2 arrays was evident, with the lowest ∆*T* occurring at the center of the arrays, approximately 24% lower compared with the average ∆*T*. Consequently, the maximum ∆*T* of 49.30 mK from the 5 × 2 array was lower than 77.05 mK from the 1 × 6 array, which had uniform coupling along the *x*-axis. For the lowest average ∆*T* case with *P* = 8 µm and *G* = 21.5 µm, Figure 8d shows weak ∆*T* distribution at the hot junctions due to destructively coupled positions in all antennas of the array. The simulation results confirmed that the optimum *P* and *G* in a finite substrate size (*S*) can enhance the *V*_oc_ in the thermoelectric nanoantenna array structure by utilizing the coupling between the antenna’s electric field and the standing wave in the substrate.

#### 3.2.3. Finite 5 × 6 Thermoelectric Nanoantenna Array

**Figure 9:**
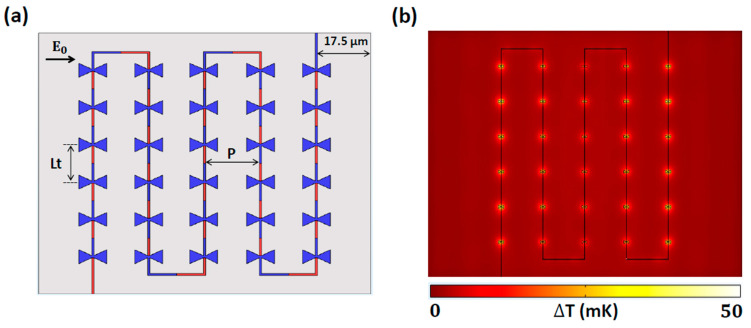
(**a**) Schematic of finite 5 × 6 arrays, consisting of 30 antennas connected in series. (**b**) Temperature distribution on the *x*–*y* plane in the middle of the antenna at 28.3 THz.

Using the optimal values for the nano-thermocouple length (*L*_t_) of 7 µm, antenna pitch (*P*) of 10 µm, and distance between the antenna and substrate boundary (*G*) of 17.5 µm, we designed a 5 × 6 finite array structure connected in series using 70 nm wide transmission lines (Figure 9a). The simulation results showed that the finite 5 × 6 arrays had an average ∆*T* of 48.37 mK. Using *V*_oc_ = Δ*S* × Δ*T*, the *V*_oc_ of the proposed structure was found to be 39.18 µV, which corresponded to an average *V*_oc_ per antenna in the array of ~1.31 µV. This value outperformed a *V*_oc_ of 1 µV from the suspended antenna over a cavity given the same input power of 1.42 W/cm^2^ [23].

To understand the heat distribution in the antennas and nano-thermocouple, the temperature distribution on the *x*–*y* plane in the middle of the antennas is presented in Figure 9b. Figure 9b shows elevated ∆*T* values of approximately 50 mK near all the antenna elements with expected variations where antennas near the boundary maintained higher ∆*T* distributions compared to those near the center. This phenomenon occurred because the standing wave pattern in the substrate for the 5 × 6 array was the same as that of the finite 5 × 2 array. The difference in ∆*T* distribution between the two arrays is quantified by the $\delta_{d}$ of the average ∆*T*. The value of the $\delta_{d}$ from the finite 5 × 6 array was 11.08 mK, which was lower than 12.19 mK from the finite 5 × 2 array. This indicated that the finite 5 × 6 array maintained more uniformly distributed temperatures, attributable to additional heat flow through transmission lines connected to both ends of the series-connected array.

**Figure 10:**
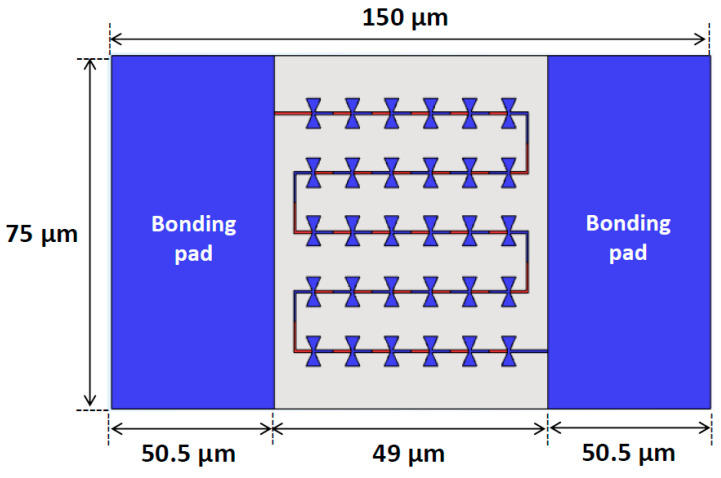
Schematic of the proposed thermoelectric antenna array with 5 × 6 antenna elements with the bonding pads.

Overall, the proposed optimization methods achieved a ∆*T* of 48.37 mK with an average *V*_oc_ of 1.31 µV per antenna. To ensure that the proposed devices generated the expected *V*_oc_ in real measurements, bonding pads composed of Ti were added to the proposed finite thermoelectric nanoantenna array, as presented in Figure 10. With the addition of 75 µm × 50.5 µm bonding pads, the overall device size became 75 µm × 150 µm. The simulation results of the 5 × 6 array with bonding pads showed that the average ∆*T* generated from all the antennas in the array was 49.60 mK, corresponding to a total *V*_oc_ of 40.18 µV and a *V*_oc_ per antenna of 1.34 µV. The bonding pads did not perturb the ∆*T* of the antenna array due to their electrically large size compared with the antenna resonant wavelength. Lastly, if a device area of ~75 × 50 µm^2^ were scaled up to 1 cm^2^ with a massive array, then a high *V*_oc_ of 1.07 V could be achieved.

### 3.3. Device’s Voltage Responsivity (β_v_) and Performance Comparison

Based on the maximum *V*_oc_ generated by the finite 5 × 6 arrays of the thermoelectric nanoantenna, we further evaluated the device’s voltage responsivity (*β_v_*), defined as the ratio between the generated *V*_oc_ and the incident power (*P_in_*) for a fair comparison to other devices. The *P_in_* of our device was 5.22 × 10^−5^ W, calculated by multiplying the effective area (*A_eff_*) of 49 × 50 µm^2^ and the laser power density (*P_d_*) of 1.42 W/cm^2^. Here, the physical substrate size where the arrays were laid out was used as the effective area. Finally, the ratio between the *V*_oc_ of 40.18 µV and the *P_in_* provided a *β_v_* of 0.77 V/W. 

A performance comparison of the proposed work with other thermoelectric nanoantenna array structures is provided in Table 1. Our structure exhibited a similar or higher *β_v_* compared to thermoelectric antenna arrays using novel metals such as Ni or Pd [22,23,40]. In [23], a suspended nanoantenna array device over an air-filled cavity exhibited a *β_v_* of 0.64 V/W, which is lower than 0.77 V/W of our device. The high *β_v_* of the proposed array device was attributed to a temperature-boosting effect from the standing wave excited in the optimized open-ended and grounded substrate, along with the bowtie antenna topology, which had a higher effective aperture compared to the counterpart dipole antennas [22,23,40]. Recent numerical studies showed that metal nanoantennas coupled with telluride-based high-ZT materials such as Bi_2_Te_3_ and Sb_2_Te_3_ achieved a much higher *β_v_* of ~50 V/W [41,42]. This difference can be mostly attributed to a higher Seebeck coefficient compared to that of novel metals. Therefore, if materials with a higher Seebeck coefficient are applied in the proposed nanoantenna array structure, the *β_v_* could be further improved.

## 4. Conclusions

We introduced a high-output voltage IR-harvesting device based on a bowtie nanoantenna array combined with a bimetal nano-thermocouple mounted on a finite SiO_2_ substrate. First, the optimal thermoelectric antenna element for achieving the maximum *V*_oc_ was found. The optimum structure was a single metal nanoantenna with low thermal conductivity among two metals used in the bimetal thermocouple, and the antenna was perpendicularly connected to the thermocouple. Subsequently, we designed a finite array structure by optimizing the nano-thermocouple length (*L*_t_), antenna pitch (*P*), and distance between the antenna and the substrate boundary (*G*). The optimal values of *L*_t_, *P*, and *G* were 7 µm, 10 µm, and 17.5 µm, respectively, in a finite 5 × 6 array with 30 thermoelectric antennas connected in series. In numerical simulations, the final thermoelectric nanoantenna array structure exhibited a ∆*T* of 49.60 mK between the hot and cold junctions, equal to a *V*_oc_ of 40.18 µV. This indicated that the average *V*_oc_ associated with one antenna was ~1.34 µV with a $\beta_{v}$ of 0.77 V/W, surpassing the previously reported values of ~1 µV and 0.64 V/W for a thermoelectric nanoantenna arrays using the same input power density of 1.42 W/cm^2^ [23]. In addition, the proposed design is expected to demonstrate better stability in real fabrication because the antenna and the nano-thermocouple are directly placed on the substrate, rather than being suspended over the air-filled cavity [23]. Consequently, the proposed optimum thermoelectric nanoantenna array design utilizing the finite and grounded SiO_2_ substrate offers a viable solution for enhancing the output voltage of IR harvesting devices and sensors.

## Figures and Tables

**Table 1:** Performance comparison of the voltage responsivity (*β_v_*) with other state-of-the-art thermoelectric nanoantenna arrays.

Ref.	Antenna Topology (Material)	Thermocouple	Substrate	# of Antennas	Power Density (W/cm^2^)	Aperture Size (µm^2^)	*V*_oc_ (µV)	*β_v_* (V/W)
[22]	Dipole (Pd)	Pd	Grounded SiO_2_	440	1.42	7.04$\;\times \;10^{3}$	3.25	0.032
[23]	Dipole (Pd)	Ni-Pd	Air-filled cavity	200	1.42	2.2$\;\times \;10^{4}$	200	0.64
[40]	Dipole (Pd)	Ni-Pd	Air-filled cavity	52	1.42	6$0\;\times \;10^{3}$	9.37	0.011
[41]	Bowtie (Au)	Bi_2_Te_3_-Sb_2_Te_3_	Air-filled cavity	16	0.1	1.1$\;\times \;10^{3}$	55	50
[42]	Dipole (Au)	Bi_2_Te_3_-Ni	Air-filled cavity	16	0.1	5.76$\;\times \;10^{3}$	30	52.08 (40 THz)
This work	Bowtie (Ti)	Ni-Ti	Open-ended and grounded SiO_2_	30	1.42	3.67$\;\times \;10^{3}$	40.18	0.77

All the *V*_oc_ and *β_v_* values are maximal at 28.3 THz if the frequency is not specified.

## Data Availability

Data are contained within the article.
